# Enhancing Breast Reconstruction with Bovine Pericardium: A Preliminary STEP (Surgical Techniques and Efficacy in Pericardium Use) Towards Improved Outcomes

**DOI:** 10.3390/jcm14176296

**Published:** 2025-09-06

**Authors:** Donato Casella, Nicola Rocco, Gianluigi Luridiana, Marco Marcasciano, Irene Zerini, Silvia Sordi, Alessandro Neri, Giuseppe Catanuto, Pietro Maria Ferrando, Juste Kaciulyte

**Affiliations:** 1 Oncologic Breast Surgery Unit, Department of Medicine, Surgery and Neuroscience, University of Siena, 53100 Siena, Italy; donatocasella@gmail.com (D.C.); irene.zerini@gmail.com (I.Z.); silvia.sordi@ao-siena.toscana.it (S.S.); neriale65@gmail.com (A.N.); 2 Department of Advanced Biomedical Sciences, University of Naples Federico II, 80138 Naples, Italy; nicolarocco2003@gmail.com; 3 Unit of Oncologic and Breast Surgery, A.R.N.A.S. Brotzu, Businco Oncologic Hospital, 09047 Cagliari, Italy; gianluigi.luridiana@aob.it; 4 Plastic and Reconstructive Surgery Unit, Department of Experimental and Clinical Medicine, “Magna Graecia” University of Catanzaro, 88100 Catanzaro, Italy; dott.marcomarcasciano@gmail.com; 5 Breast Unit, University Hospital Federico II, 80131 Naples, Italy; giuseppecatanuto@gmail.com; 6 Plastic Surgery Department, AOU Città della Salute e della Scienza di Torino—CTO Hospital, 10126 Torino, Italy; pmferrando@hotmail.it

**Keywords:** breast cancer, breast reconstruction, acellular pericardium matrix, peri-prosthetic device

## Abstract

**Background/Objectives**: In pre-pectoral breast reconstruction, both synthetic meshes (SM) and acellular dermis or pericardium matrixes (ADM/APM) present drawbacks that can be prevented with targeted device choosing. In daily practice, the authors wrap the implant with a human-derived ADM (hADM) when they found mastectomy flaps thinner than 1 cm. When hADM is not available, an APM is used. Here, the authors present their results with APM utilisation. **Methods**: From January to September 2024, patients undergoing pre-pectoral breast reconstruction with mastectomy flaps thinner than 1 cm were selected. Specifically, implants were wrapped in bovine pericardium (Exaflex—MAGGI Srl, TO, Italy). During a minimum 6 months follow-up, outcomes were recorded; in particular, rippling incidence was assessed with indication for secondary fat grafting. **Results**: Nineteen patients met the inclusion criteria. Average age was 54.4 years (range: 39–70), three of them were smokers (15.8%) and three were affected from diabetes or hypertension (15.8%). With one case bilateral, a total of 20 mastectomies were performed. Intra-operative mastectomy flaps’ thickness mean measure was 0.75 cm (range: 0.5–0.8). All of them underwent two-stage pre-pectoral IBR with APM covering the implant. During a mean follow-up of 9.5 months (range: 6–13), no major post-operative complications occurred and five cases (25%) presented rippling. **Conclusions**: With no consensus on the superiority of either biologic matrixes or SMs, the authors extended their patients’ selection tool to aid in the choice of peri-prosthetic device. The bovine APM use showed capacity of reducing secondary lipofilling interventions in patients with thin mastectomy flaps.

## 1. Introduction

Implants have been used in breast reconstruction since the 1960s, when the pre-pectoral approach was first introduced, aiming to respect the gland’s anatomic position [1]. This early experience in pre-pectoral reconstruction was not fortunate due to poor materials’ quality and excessively thin mastectomy flaps [2,3]. After a long domination of submuscular implant placement, pre-pectoral breast reconstruction came back into clinical practice in the 21st century. Today, pre-pectoral reconstruction technique is celebrated for its anatomical fidelity, reduced intra-operative trauma, and faster recovery. However, its success hinges on meticulous patient selection and the strategic use of peri-prosthetic devices to counteract complications such as rippling, implant malposition, and capsular contracture.

The reintroduction of pre-pectoral implant-based breast reconstruction (IBR) represents a convergence of surgical ingenuity and biomaterial science and is a consequence of fundamental evolutionary steps in oncologic breast surgery. Mastectomy techniques were refined, becoming more conservative: skin-sparing and nipple–areola-sparing mastectomies (SSM and NASM) were described in the 1980s [4] and ensured oncological safety [5,6] together with breast envelope preservation [7]. Breast implants evolved as well, transitioning from smooth to textured surfaces, anatomical shaping, and cohesive gel fillers with improved stability and natural aesthetics [8]. Finally, the crucial event for pre-pectoral reconstruction revival was the introduction of peri-prosthetic devices such as synthetic meshes (SM) [9] and acellular dermal matrixes (ADM) that addressed historical pitfalls by reinforcing thin mastectomy flaps [10]. These devices are wrapped around the implant to improve its coverage, they allow the control of its positioning in the subcutaneous pocket, and they significantly reduce common complications associated to pre-pectoral reconstruction such as rippling and capsular contracture [11,12]. Both SM and ADM categories are not exempt from drawbacks. SMs are typically composed of polypropylene or titanium-coated polymers. They offer tensile strength and cost-effectiveness; however, their rigidity and foreign body response correlate with higher seroma formation and increased risk of rippling, in patients with low BMI and thin mastectomy flaps, in particular. Rippling is defined as the pre-pectoral implant’s visibility as palpable folds or wrinkles in the upper and medium quadrants. Currently, its incidence is reported to lie between 0 and 35%. In 2019, Vidya et al. [13] described a grading system for rippling in an attempt to give objective indications for its treatment. According to their system, grade 1 rippling represents the lowest grade and corresponds to the absence of rippling evidence seen both at rest and with movement. The highest grade (grade 4) is defined as severe persistent rippling that creates gross deformity both at rest and with movement. This aesthetic issue can be easily addressed with one or few fat grafting procedures [14] or it can be prevented with an accurate patients’ selection and targeted indication for specific reconstructive strategy and device choosing. In cases at risk for the implant’s visibility and rippling, such as those undergoing breast revision surgery, following radiotherapy or presenting thin skin coverage, ADMs have been recommended instead of SMs [15]. Being soft de-cellularised tissue grafts, ADMs act as a scaffold for the surrounding tissues’ cells and promote host cell colonisation and vascularisation. On the other side, ADMs have been associated with higher costs and complications such as Red Breast syndrome, seroma, and infection [16].

Given this premise, it appears clear that surgeons practicing pre-pectoral breast reconstruction with ADMs and SMs, face the choice of a wide range of products. To date, there is no comprehensive identification of their proper applications in the literature.

The authors of the current study apply the Pre-Bra score [17] to select the most suitable implant-based breast reconstructive technique for each patient. For those elected for direct-to-implant (DTI) or temporary expander-based (TE) pre-pectoral reconstruction and presenting mastectomy flaps thinner than 1 cm, they wrap the implant with DED (de-epidermised dermis), a cadaver-derived ADM (hADM) provided by the Regional Skin Bank of Siena. In addition to breast surgery, hADM has proven its efficiency in several surgical fields such as burns and wound management, hernia repair in general surgery, or reconstructive gynaecology [18]. Beyond its recognised efficiency and the benefit in terms of significant cost reduction [19], DED presents a substantial boundary: its disposal is limited to the quantities present in the tissue bank at the moment of the surgery. As a solution to this obstacle, the authors started to use an acellular pericardium matrix (APM), called Exaflex (MAGGI Srl, Andezeno, TO, Italy), as a cost-effective ($1.200–$2.000 per sheet) and biocompatible alternative. Its bilayered structure—combining collagen-rich layers for durability and elastin for flexibility—mimics the human dermis, offering a promising solution for high-risk patients [20,21].

In the current multicentric preliminary study, the authors present their results in using this APM in a specifically selected group of patients undergoing immediate pre-pectoral IBR.

## 2. Patients and Methods

Patient selection for the current study started from January 2024 at the Breast Unit of Azienda Ospedaliera Universitaria Senese in Siena, Italy. Main inclusion criteria were age above 18 and breast cancer diagnosis that required mastectomy in patients eligible for immediate, pre-pectoral IBR.

Preoperative patients’ assessment was performed with the Pre-Bra score in order to select the patients feasible for pre-pectoral breast reconstruction [17].

Thanks to an accurate dissection, SSM/NASM procedures preserved the inframammary fold and lateral mammary sulcus. An intra-operatory implementation of the Pre-Bra score added an adjunctive evaluation of the mastectomy flaps’ thickness with a sterile millimetre-graded ruler. The flap thickness represented an independent variable that guided surgeons in choosing which peri-prosthetic device to use. To date, in the literature, there is no proof of any peri-prosthetic device superiority for IBR [22,23,24]. Nevertheless, a recent study showed the potential capacity of ADM/APM devices in reducing the incidence of rippling after pre-pectoral IBR in patients with thin mastectomy flaps [25]. Following these data, only cases eligible for two-stage pre-pectoral IBR (Pre-Bra score: 5–8) with flaps thinner than 1 cm were admitted in the current study.

**Figure 1:**
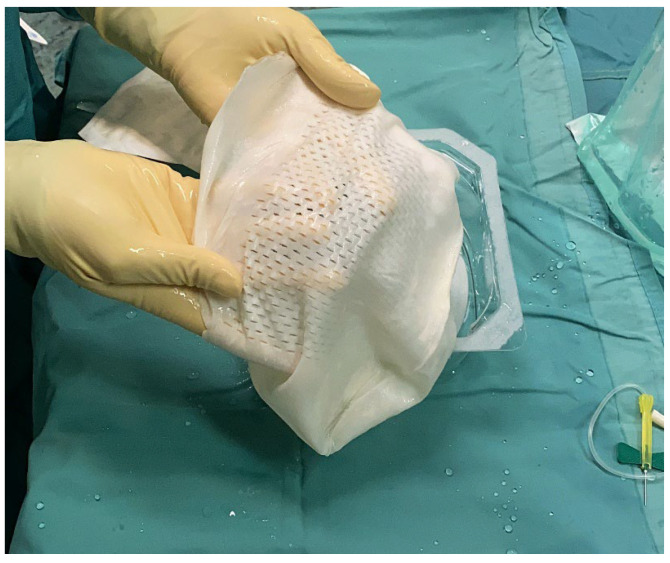
Intra-operative picture that shows the bilayered bovine pericardium matrix (APM) rehydrated in saline and ready to cover the implant.

**Figure 2:**
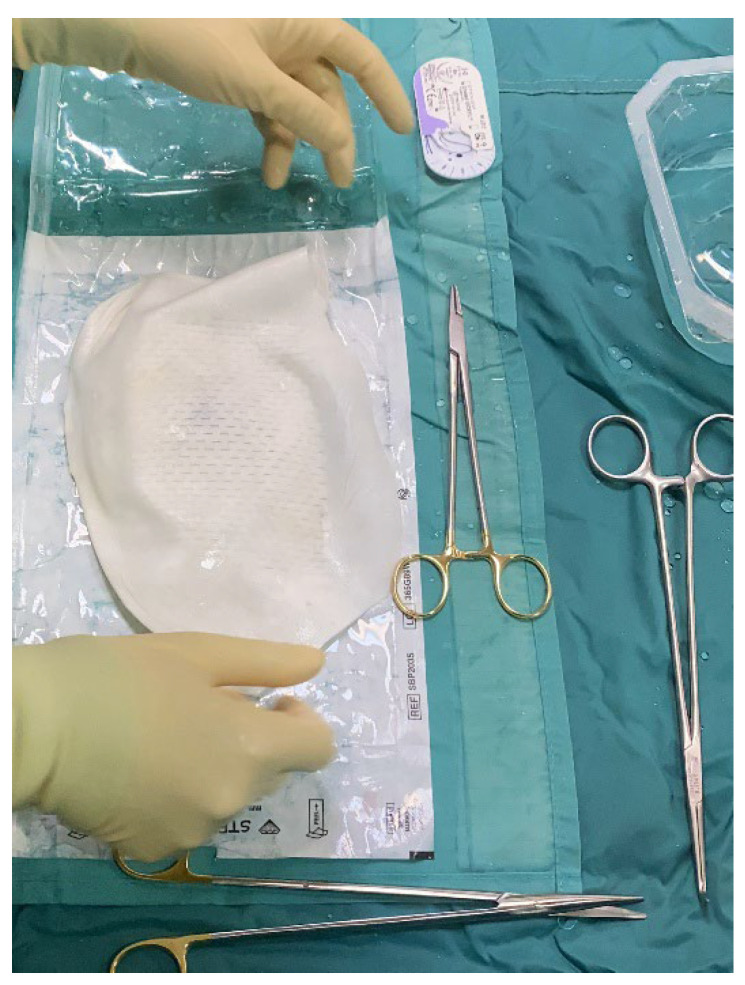
The intra-operative picture shows the APM covering the tissue expander, already filled with saline solution up to 60% of its volume. The APM is going to be trimmed to match implant dimensions and closed around it using 2-0 resorbable braided stitches.

Specifically, implants were wrapped in bilayered bovine pericardium (Exaflex) (Figure 1 and Figure 2). The APM was rehydrated in saline for 15 min, trimmed to match implant dimensions, and sutured to the pectoral fascia using 2-0 Vicryl (Ethicon, Cincinnati, OH, USA). Tissue expanders were filled to 60% capacity to minimise tension on flaps. One 19-Fr blake drain per breast was placed subcutaneously and removed when output fell below 30 mL/day. In all cases, intra-operative indocyanine green (ICG)-based fluorescent angiography exam was performed to evaluate mastectomy flaps’ perfusion. Ambulatory expansions started at the third post-operative day and every 15 days following, reaching 100% of expanders’ volume in an average of 45 days after 3 to 5 expansions. The mean final volume of the expanders was 455 mL (ranging from 350 to 650 mL). There were no impediments to the expansion thanks to the fenestration of the APM that gives the device elastic properties. All the expanders presented the valve in the upper pole, which was detected with magnetic or radio-frequency-based locators prior to every expansion. The timing for tissue expander replacement with definitive implant was scheduled based on eventual chemotherapies or radiotherapies, post-operative complications, and personal patient’s preference. The average time between the first and second surgery consisted of 6.7 months, with a range that spanned from 4 to 13 months. During the second surgery, the same skin incision was used. The APM was found fully resorbed into a smooth and malleable peri-prosthetic capsule that was incised to exchange the expander with the definitive implant and closed afterwards with resorbable stitches. All the definitive implants (Mentor^TM^, CPG^TM^ Gel Breast, Irvine, CA, USA) used in the current series presented a teardrop shape with low (85%) or mild (15%) projection. Their volume ranged from 300 to 615 mL (average, 419.2 mL).

During a minimum 6 months follow-up, post-operative outcomes including early and late complications were recorded at 1, 3, 6, and 12 months. In particular, rippling incidence with consequent indication for secondary fat grafting was assessed at the 6th month follow-up visit by two plastic surgeons that did not perform the surgeries.

## 3. Results

From January to September 2024, 19 patients undergoing mastectomy and immediate IBR that presented mastectomy flaps thinner than 1 cm and a Pre-Bra score between 5 and 8 were selected for the current study (Table 1).

Their average age was 54.4 years old (range: 39–70) and their body mass index spanned from 19 to 25 kg/m^2^ (average: 22 kg/m^2^). Three of them were active smokers (15.8%), one was affected from diabetes (5.3%), and two patients presented hypertension (10.5%). Six patients (31.6%) presented breast ptosis with indication for skin-reducing mastectomy (SRM), while 36.8% of them (seven cases) had undergone omolateral breast surgery previously and two (10.5%) had received radiotherapy as well. Pre-operative assessment with the Pre-Bra score ranged between 6 and 8 (average: 7.2) and intra-operative mastectomy flaps’ thickness mean measure was 0.75 cm (range: 0.5–0.8 cm). These characteristics posed indication for two-stage pre-pectoral IBR with TE coverage with APM (Table 2).

With one case bilateral, a total of 20 mastectomies were performed, of which 6 (30%) were SRM while the rest were SSM or NASM (14, 70%). Intra-operative ICG-based angiography confirmed the vitality of mastectomy flaps in all cases and the perfusion of the nipple–areola complex (NAC) in the NASMs and in the SRMs. Drains were kept for an average of 6.5 post-operative days (range: 3–11 days).

**Figure 3:**
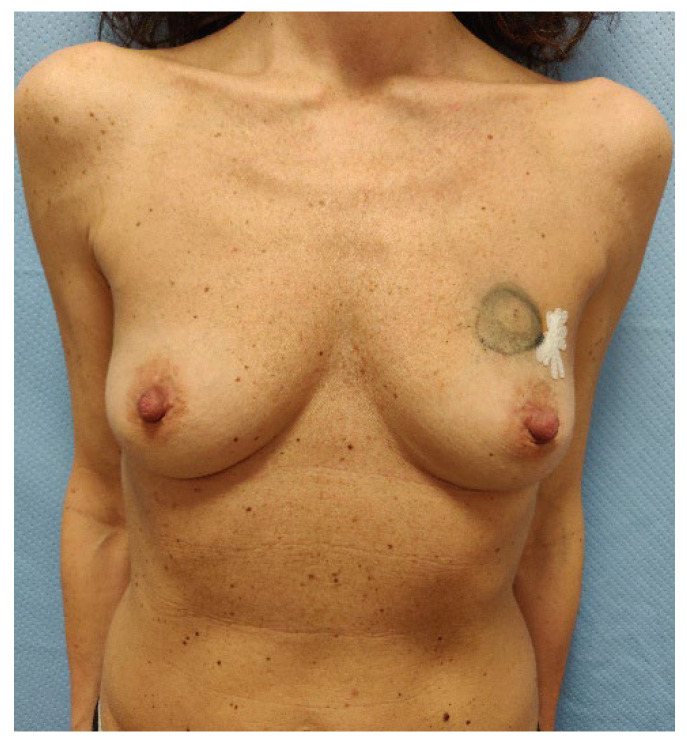
The picture shows pre-operatory assessment of the case 1 patient, a 45-year-old woman that presented with an invasive ductal breast carcinoma in the upper-central quadrant of her left breast. The patient had no comorbidities and was a former smoker. She collected 7 points at the Pre-Bra score evaluation and was scheduled for NASM, sentinel lymph node biopsy, and two-stage IBR.

**Figure 4:**
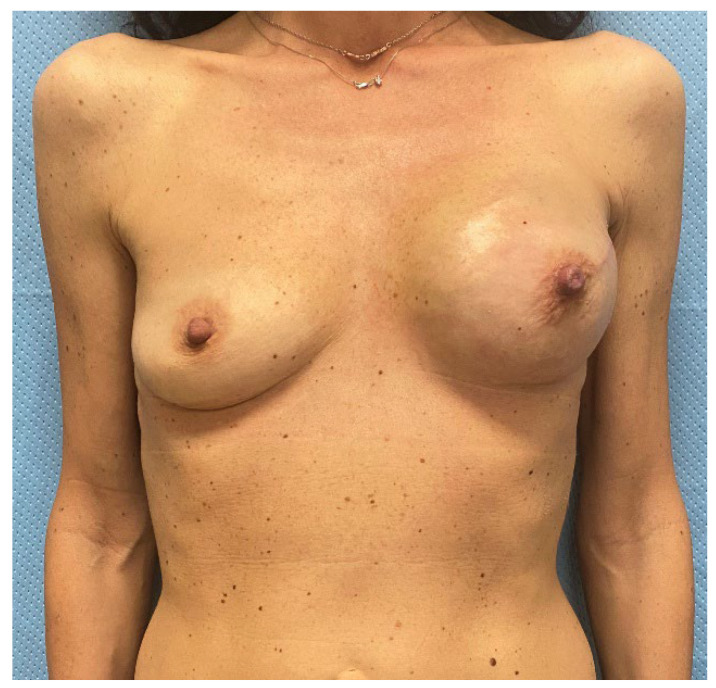
At the 1 month follow-up visit, the case 1 patient presented complete wound healing. During surgery, the mastectomy flaps were well perfused yet thinner than 1 cm, so the tissue expander was covered with APM before being placed in the pre-pectoral pocket.

**Figure 5:**
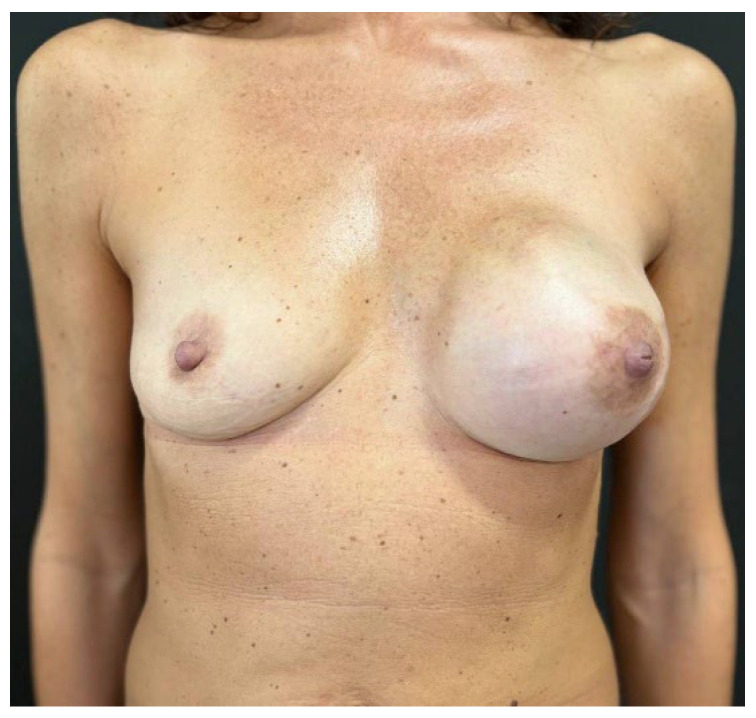
Case 1 patient at the 3 month follow-up visit: the tissue expander has been filled up to 100% of its volume and the patient is ready for the second breast reconstructive surgery.

**Figure 6:**
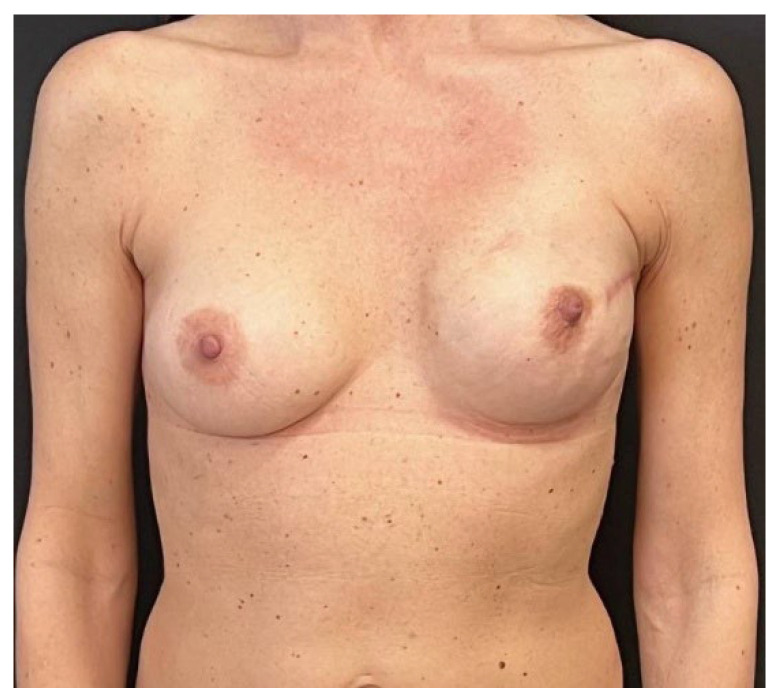
Three months after tissue expander substitution with definitive implant and a contralateral breast augmentation with retroglandular implant, the case 1 patient shows good breast symmetry and no signs of rippling.

**Figure 7:**
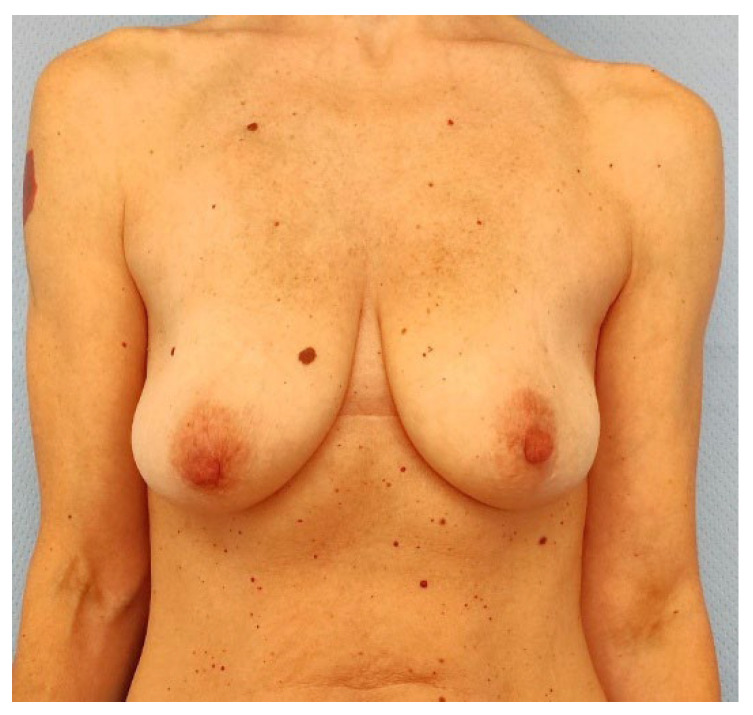
Preoperatory picture of case 2 patient, a 43-year-old female that presented with an extended (65 × 45 mm) left breast cancer affecting the upper lateral quadrant. The patient presented no comorbidities and no previous breast surgeries but was a smoker and her mastectomy flaps were thinner than 1 cm. Her Pre-Bra score resulted in a 7, so she underwent SSM, sentinel lymph node biopsy, and two-stage IBR with APM coverage over the tissue expander.

**Figure 8:**
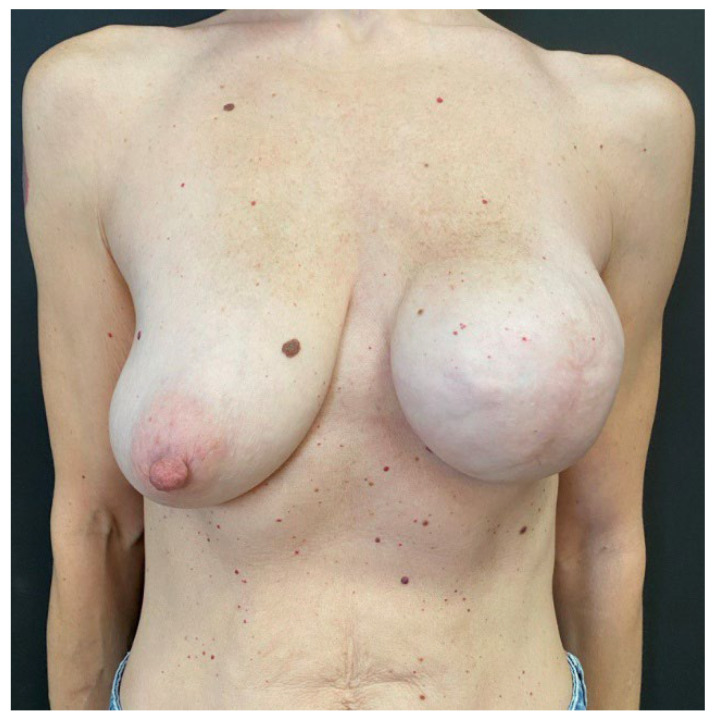
At the 6 month follow-up visit, the case 2 patient showed full device expansion and was scheduled for its substitution with definitive implant and a contralateral breast mastopexy.

Mean follow-up consisted of 9.5 months (range: 6–13 months) and no major post-operative complications occurred (Figure 3, Figure 4, Figure 5, Figure 6, Figure 7 and Figure 8). Only 2 cases out of 20 mastectomies (10%) presented minor complications that were successfully managed in the outpatient ambulatory within the first 30 post-operative days (Table 3).

From the 6th month follow-up visit, 5 cases (25%) presented rippling in the upper-inner quadrants and a secondary fat grafting procedure has been proposed to these patients. Two of them did not wish to proceed with the corrective surgery, having no pain and declaring satisfaction with the result despite the rippling observed by the surgeons. The 3 cases that underwent secondary fat grafting achieved aesthetic improvement and satisfaction after a single procedure that transferred approximately 50 mL of autologous fat.

## 4. Discussion

Since their introduction, peri-prosthetic devices became paramount to pre-pectoral IBR success, together with an accurate patients’ selection and targeted surgery for each case. Both biologic matrixes and SMs can be placed over the anterior implant’s surface as a reinforcement or they can wrap the implant entirely. ADMs were first employed in submuscular breast reconstructions in 2006 [26]. Thanks to advantages in terms of capsular contracture and implant placement control, soon they gained major role in pre-pectoral breast reconstruction, even if their wrapping around the implant is an off-label procedure in the US, to date. All ADM/APMs in use nowadays are animal-derived (bovine or porcine) or human-derived (hADM). The animal-derived ones are widespread in the world, despite their average being five thousand US$ upwards per breast, on average [27]. On the other side, hADMs commerce is forbidden by European legislations and they can only be provided as dermal allografts by tissue establishments authorised by competent authorities. Adjunctive limits to the hADMs spread among surgeons are given by the need for longer rehydration and mandatory side-orientation of some of these devices, or their discouraged use in patients with autoimmune connective tissue disorders [28]. The main limit met by the authors of the current paper is the hADM availability that is strictly linked to the quantities of product at disposal in the tissue bank.

As per the various animal-derived ADMs, they did not show significant differences, among various models, in post-operative complications incidence after pre-pectoral DTI breast reconstruction, according to a recent systematic review [29] that sorted the ADM devices into three groups based on their origin: bovine, porcine Braxon^®^ (Decomed SrL, Venezia, Italy) where the prosthesis was fully wrapped, and other porcine ADMs.

The first ADMs used in pre-pectoral breast reconstruction were porcine-derived, as they have been reported to have a low major complication rate [30]. This preliminary experience that supported safe application of porcine ADMs suggested similarities in the performance of other animal-derived matrices and opened the way to develop bovine APMs. Bovine pericardium matrix was analysed and its safety was supported in IBR despite reports of complications that included flap ischemia and rippling. Its performance aligns with porcine ADMs in rippling reduction (25% vs. 20–30%) but surpasses SMs (35–45%). Moreover, the bilayered structure of bovine APM may enhance mechanical support, particularly in thin-flap patients, by distributing lateral tension across collagen fibres. Histologic studies further suggested bovine pericardium’s rapid vascularisation, with capillary ingrowth occurring within 4–6 weeks versus 8–12 weeks for porcine ADMs, potentially lowering infection risks [31,32]. It must be stated that the use of bovine-derived materials necessitates transparent patient counselling, particularly regarding cultural/religious sensitivities. In this cohort, no patients declined bovine APM use after education on tissue sourcing and safety protocols.

Already, in 2013, Gaster et al. [33] conducted a study that focused on the histologic analysis of foetal bovine-derived ADM in breast reconstruction. Their findings indicated potential suitability for bovine-derived matrixes use in humans, for their minimal inflammatory response and good integration in surrounding tissues. Later on, bovine APMs have been associated to specific complications such as flap ischemia, hematoma, and marginal skin flap necrosis, but also with low rates of capsular contracture [31]. In general, the presence of complications was higher in patients who had undergone radiotherapy or therapeutic mastectomy. The significance of these findings appears multifaceted. On one hand, low inflammatory response and satisfactory integration into host tissues promote the use of bovine pericardium matrices, even if the association with certain complications emphasises the need for proper patient selection and consideration of individual risk factors.

Since 2014, the current paper’s authors showed safety and feasibility of pre-pectoral implant placement when wrapped into a non-resorbable titanium-coated polypropylene SM [34].

If compared to their biologic counterpart, SMs offer good strength and pliability at lower prices, even if they require a significant learning curve from the surgeons that approach them for the first time [35]. SMs are flexible absorbable, partially absorbable, or non-absorbable sheets that provide a complete implant envelope and act like an additional layer over the implant, stimulating the formation of a sort of “neo-fascia” [36]. One of the major drawbacks they observed in the last decade, especially in cases with thinner mastectomy flaps, is the consistent incidence of rippling phenomenon at the upper and medial poles, in particular. Rippling and implant edge visibility occur in an average of 12.9–19.4% of pre-pectoral IBRs and fat grafting may mitigate this condition, so it is mandatory to counsel the patients by explaining to them the possibility of ulterior surgical procedures need [37].

As a response, the authors applied their experience with the Pre-Bra assessment score to select which cases may benefit from biologic matrixes use instead of SM. For the current study, 19 patients showed the characteristics to be selected, being candidates to mastectomy and following IBR. More specifically, according to the authors’ clinical practice, patients that score high or medium results with the Pre-Bra system are elected for pre-pectoral DTI or temporary TE placement, respectively. Intra-operatively, mastectomy skin flaps’ vitality and thickness are assessed with ICG-based fluorescent angiography. Cases with vital flaps but reduced thickness (<1 cm) undergo pre-pectoral breast reconstruction with implant coverage with APM, specifically Exaflex in the current study. Exaflex is part of a new category of biologic meshes made of bilayered bovine pericardium that made their first appearance in breast reconstructive surgery in 2011, after an initial successful use in hernia repair surgeries [38]. Their collagen type I three-dimensional structure gives great resistance to tensile forces, while their fenestration and thin profile facilitate fluid outflow away from the implant while ensuring an anti-inflammatory effect in the pocket. The bovine APM were proven to be as safe as other biologic matrixes yet with lower costs [20,21]. These characteristics drove the authors to use this APM when rippling prevention was indicated [13,39], yet no hADM is at disposal. Exaflex, in particular, presents a bilayered structure where the porous fibrillary layer promotes early neo-angiogenesis and fast cell ingrowth while the compact layer provides structural support being rapidly populated by patients’ fibroblasts [40].

The current preliminary study investigated overall reconstructive results and rippling incidence, in particular, after 20 mastectomies followed by two-stage pre-pectoral IBR with TE covered with APM. The 19 patients were selected accurately. Despite seven of them being active or past smokers, and the fact that three presented uncontrolled diabetes or hypertension and two had undergone previous radiotherapy, none of these single characteristics were considered a contraindication for implant positioning. Global evaluation of each case showed indication for two-stage pre-pectoral breast reconstruction. Of the 20 mastectomies performed, 6 were performed in a skin-reducing pattern and an inferior dermal flap allowed us to add coverage to the implant and the APM.

Intra-operatory evaluation allowed surgeons to detect cases with major risk for rippling that presented mastectomy flaps thinner than 1 cm. In the current series, average flap thickness was 0.75 cm, spanning from 0.5 to 0.8 cm. Indeed, rippling phenomenon is characterised by palpable or visible folds on the surface of the reconstructed breast, especially in the upper and medial quadrants. This aesthetic issue can be related to lack of overlying tissue support, thin-skin soft tissue mantles, and redistribution of subdermal adipose tissue, with low BMI, multiple breast surgeries, revision surgeries, neoadjuvant chemotherapy, and low cohesive breast implants being possible risk factors [41,42]. In the presence of these risk factors, there are some options to prevent rippling that have been described in the literature, such as the use of ADM coverage, fat grafting, and other techniques like implant upsizing or preparing a small pocket [39]. More invasive preventive methods have been described as well, like the superior coverage technique that was introduced by Pittman et al. [43] in an effort to reduce rippling deformities after pre-pectoral breast reconstruction. Similarly, Ryu et al. [44] reported the P1 technique that consists of harvesting a pectoralis muscle slip to cover the upper poles after complete wrapping of the implant with ADM.

After a minimum of 6 months follow-up (average, 9.5 months), 5 patients (25% of the operated breasts) of the current study presented rippling that required a secondary corrective fat grafting. The phenomenon was localised in the upper-inner quadrants mostly, with no pain nor capsule contracture associated. Only three of those patients agreed with the secondary procedure indication and underwent a single transfer of 50 mL of autologous fat that managed to correct the aesthetic issue. The other two did not agree with the surgeons’ evaluation and were satisfied with the reconstruction as it was. This patients’ preference may be explained with the main advantages that characterise a successful pre-pectoral implant-based breast reconstruction: a fast recovery with low post-operative pain and no impact on upper limb’s function or movement. All these factors allow the patient to return to her daily activities early, thus improving her quality of life and probably giving less focus to minor aesthetic imperfections such as rippling phenomenon. Nevertheless, for those patients that do present concerns about the aesthetic outcomes of their pre-pectoral breast reconstruction, autologous fat grafting represents an effective adjunct technique that addresses common issues such as implant rippling, visibility, and irregular contours [45]. In the current series, no major surgical interventions such as implant exchange or pocket conversion were needed to address rippling.

Thanks to the accurate selection system developed by the authors, no major post-operative complications occurred. Only two patients developed minor complications (one early seroma and one wound dehiscence) that were managed in the outpatient setting, with no need for re-operation. Moreover, in a population that presented high risk for rippling, the incidence of this unpleasant and bothersome outcome was contained to 25%, in line with numbers reported in the literature [13] and consistently lower when compared to authors’ experience with SM use in patients with similar characteristics [25].

Important limits of the current study must be considered. First, the small size of the population with no power calculation limits the robustness of the results. Even more, the small selection of population with limited complex cases is scarcely representing the general population. Similarly, the lack of a control group with no comparison between different ADM/APMs or SMs affects the significance of the results described. A major limitation is represented by the brief follow-up period that may be too short to detect late-onset complications such as capsule contracture that requires a minimum of 24 months for an accurate estimation. Finally, observer bias may have been caused by the lack of validated tools like the BREAST-Q, in assessing post-operative rippling. The same bias could derive from the operator-depended intra-operatory assessment of the mastectomy flaps that is subject to intra- and inter-observer variability, thus reducing the study reproducibility.

In the future, studies with larger and multicentre cohorts and longer follow-up may allow better outcomes evaluation such as implant integrity, aesthetic results, patient satisfaction, and complication rates over time. All this is with the aim to corroborate the current preliminary findings in order to validate the patients and reconstructive pathway selection described. As innovation accelerates, the integration of bioengineered scaffolds and machine learning-driven predictive models will further refine outcomes, cementing pre-pectoral reconstruction as a gold standard in breast oncology.

## 5. Conclusions

In order to reduce complications and failures, an accurate patients’ selection is paramount to target the most suitable breast reconstructive path for each case. With no consensus on the superiority of either biologic matrixes or SMs, to date, the authors extended their patients’ selection tool to aid in the choice of which peri-prosthetic device type to apply. In cases at risk to develop rippling for thin mastectomy flaps, the APM showed capacity of reducing secondary lipofilling interventions, thus giving indication for their use in these specific occurrences. The bovine APM used in the current study showed cost-efficacy and biocompatibility, thus addressing global disparities in reconstructive access, particularly in resource-limited settings.

## Figures and Tables

**Table 1:** Baseline characteristics and pre-operative assessment of the 19 patients selected.

Characteristics	Value (%)
No. of patients	19
Age, yr	
Mean	54.4
Range	39–70
BMI Kg/m^2^	
Mean	22
Range	19–25
Smoking	
Active smokers	3 (15.8)
Ex-smokers	4 (21)
Never smokers	12 (63.2)
Not-controlled Diabetes	1 (5.3)
Not-controlled Hypertension	2 (10.5)
Previous Radiotherapy	2 (10.5)
Previous Breast Surgery	7 (36.8)
Breast Ptosis (grade II or III)	6 (31.6)
Pre-Bra Score	6–8 (7.2)

BMI, Body Mass Index.

**Table 2:** Twenty surgical procedures performed on 19 patients during the study.

Characteristics	Value (%)
Total number of mastectomies	20
monolateral	18 (90)
bilateral	2 (10)
Type of mastectomy	
SSM	5 (25)
NASM	9 (45)
SRM	6 (30)
Type of pre-pectoral breast reconstruction	
Two-stage with TE	20 (100)
Axillary surgery	
SNB	19 (95)
Mastectomy flaps’ thickness	
Average	0.75 cm
Range	0.5–0.8 cm

SSM, skin-sparing mastectomy. NASM, nipple–areola-sparing mastectomy. SRM, skin-reducing mastectomy. TE, tissue expander. SNB, sentinel node biopsy.

**Table 3:** Post-operative complications that were registered during an average 9.5 month follow-up.

	Value (%)
Early complication	2 (10)
Seroma	1 (5)
Surgical wound dehiscence	1 (5)
Hematoma	0
Infection	0
Late complication	5 (25)
Rippling	5 (25)

## Data Availability

The original contributions presented in this study are included in the article. Further inquiries can be directed to the corresponding author.

## Abbreviations

The following abbreviations are used in this manuscript:

IBR	Implant-based Breast Reconstruction
SSM	Skin-Sparing Mastectomy
NASM	Nipple–Areola-Sparing Mastectomy
SM	Synthetic Mesh
ADM	Acellular Dermal Matrix
DTI	Direct-To-Implant
TE	Temporary Expander
DED	De-Epidermised Dermis
hADM	human cadaver-derived Acellular Dermal Matrix
APM	Acellular Pericardium Matrix
ICG	IndoCyanine Green
SRM	Skin-Reducing Mastectomy
NAC	Nipple–Areola Complex
